# Structural Comparison and Drug Screening of Spike Proteins of Ten SARS-CoV-2 Variants

**DOI:** 10.34133/2022/9781758

**Published:** 2022-02-01

**Authors:** Qiangzhen Yang, Xuemin Jian, Ali Alamdar Shah Syed, Aamir Fahira, Chenxiang Zheng, Zijia Zhu, Ke Wang, Jinmai Zhang, Yanqin Wen, Zhiqiang Li, Dun Pan, Tingting Lu, Zhuo Wang, Yongyong Shi

**Affiliations:** ^1^Bio-X Institutes, Key Laboratory for the Genetics of Developmental and Neuropsychiatric Disorders (Ministry of Education), Shanghai Jiao Tong University, 1954 Huashan Road, Shanghai 200030, China; ^2^Biomedical Sciences Institute of Qingdao University (Qingdao Branch of SJTU Bio-X Institutes), Qingdao University, Qingdao 266003, China; ^3^Shanghai Key Laboratory of Psychotic Disorders, Shanghai Mental Health Center, Shanghai Jiao Tong University School of Medicine, Shanghai 200030, China; ^4^Shanghai Key Laboratory of Sleep Disordered Breathing, Shanghai Jiao Tong University Affiliated Sixth People's Hospital, Shanghai, China; ^5^The First Affiliated Hospital of Zhengzhou University, Zhengzhou 450052, China; ^6^Department of Psychiatry, First Teaching Hospital of Xinjiang Medical University, Urumqi 830046, China

## Abstract

SARS-CoV-2 (severe acute respiratory syndrome coronavirus 2) has evolved many variants with stronger infectivity and immune evasion than the original strain, including Alpha, Beta, Gamma, Delta, Epsilon, Kappa, Iota, Lambda, and 21H strains. Amino acid mutations are enriched in the spike protein of SARS-CoV-2, which plays a crucial role in cell infection. However, the impact of these mutations on protein structure and function is unclear. Understanding the pathophysiology and pandemic features of these SARS-CoV-2 variants requires knowledge of the spike protein structures. Here, we obtained the spike protein structures of 10 main globally endemic SARS-CoV-2 strains using AlphaFold2. The clustering analysis based on structural similarity revealed the unique features of the mainly pandemic SARS-CoV-2 Delta variants, indicating that structural clusters can reflect the current characteristics of the epidemic more accurately than those based on the protein sequence. The analysis of the binding affinities of ACE2-RBD, antibody-NTD, and antibody-RBD complexes in the different variants revealed that the recognition of antibodies against S1 NTD and RBD was decreased in the variants, especially the Delta variant compared with the original strain, which may induce the immune evasion of SARS-CoV-2 variants. Furthermore, by virtual screening the ZINC database against a high-accuracy predicted structure of Delta spike protein and experimental validation, we identified multiple compounds that target S1 NTD and RBD, which might contribute towards the development of clinical anti-SARS-CoV-2 medicines. Our findings provided a basic foundation for future in vitro and in vivo investigations that might speed up the development of potential therapies for the SARS-CoV-2 variants.

## 1. Introduction

Coronavirus disease 2019 (COVID-19) outbreak began in December 2019 and has caused more than 4.8 million deaths, according to the statistics of the World Health Organization (WHO), as of October 15, 2021 (https://www.who.int/). COVID-19 is caused by severe acute respiratory syndrome coronavirus 2 (SARS-CoV-2), a positive-sense RNA betacoronavirus belonging to the family Coronaviridae [1, 2]. SARS-CoV-2 possesses a large genome of approximately 30 kb [3], which encodes for four structural proteins, spike (S), envelope (E), membrane (M), and nucleocapsid (N) proteins, and sixteen nonstructural proteins (Nsp 1-16) [4–6]. Among these proteins, the S protein plays an important role in binding the angiotensin-converting enzyme 2 (ACE2) of the host cell, which helps the virus to enter the host cell [7]. The S protein can be recognized by and bond with the cell surface toll-like receptor 4 (TLR4), as well as antibodies, so it is a target for immunological recognition [8, 9].

All viruses, including SARS-CoV-2, change over time. Although the evolutionary rate of SARS-CoV-2 is low, which displays a change of 1 or 2 nucleotides per month per lineage in the 30 kb pairs [10], a long-time and extensive spread of SARS-CoV-2 have induced some unexpected mutations that can increase virus transmission and disease severity [11–13]. So far, the worldwide spreading variants of SARS-CoV-2 are Alpha, Beta, Gamma, Delta, Epsilon, Kappa, Iota, Lambda, and Mu (21H) named by the WHO. The WHO classifies the variants of Alpha, Beta, Gamma, and Delta to variants of concern (VOC) [14–18]. Previous studies demonstrated that the Delta variant decreased the effectiveness of vaccines and increased the breakthrough infection rates [19, 20]. Many researchers have focused on developing anti-SARS-CoV-2 drugs and found some potential drugs, such as Azvudine [21], Molnupiravir [22], Paxlovid, and antibodies [23, 24].

The mutations on the S proteins have been reported to affect both the binding affinity with ACE2 and the efficacy of antibodies [12, 25–27]. Moreover, the S protein and its parts are important for designing most approved vaccines, and thus, the mutations on the S protein raised much more concern about the vaccine effectiveness of SARS-CoV-2 [12, 28–31]. Many researchers have focused on exploring the S protein structures of different SARS-CoV-2 variants by experimental and modeling methods, which has contributed immensely to our understanding of how the mutations alter the structure and function of the S protein [32–35]. However, due to rapidly increasing variants, it is still a challenge to reveal the S protein structures of all SARS-CoV-2 variants. AlphaFold2 (AlphaFold) is a computational approach capable of predicting protein structures with high accuracy, which provides us with a new method to quickly predict protein structures according to their genetic sequences. Utilization of this powerful approach can help us to solve the challenge of revealing the S protein structures of different SARS-CoV-2 variants.

In this study, we used the AlphaFold to model the spike protein structures of ten SARS-CoV-2 variants. The high-accuracy structures were verified by the comparison with experimental structures and the pLDDT (the predicted local-distance difference test) of the AlphaFold built-in algorithm. To classify the SARS-CoV-2 strains, we performed phylogenetic analyses based on the genomic and protein sequences of all strains. Moreover, we analyzed the binding affinities of ACE2 and antibodies to S1 RBD and NTD, revealing that the mutations on the Delta variant S protein could affect the recognition of antibodies with S1 RBD and NTD, which might increase the immune evasion. Furthermore, we identified multiple compounds that target S1 NTD and RBD by virtual screening the ZINC database and experimental validation, which might contribute towards the development of clinical anti-SARS-CoV-2 medicines. Our results provided abundant basic data for further research related to preventing and curing COVID-19.

## 2. Results

### 2.1. Analyses of the Sequence Mutations on Different SARS-CoV-2 Strains

SARS-CoV-2 has changed as it has spread across the globe and has evolved many variants, such as Alpha, Beta, Gamma, Delta, Kappa, Lambda, Gamma, Iota, and 21H variants. To comprehensively understand the characteristics of these SARS-CoV-2 variants, we analyzed nucleotide mutations on all SARS-CoV-2 variants. The results showed that the nucleotide mutations occur across the whole genome, including open reading frames (ORFs), spike (S) glycoprotein, nucleocapsid phosphoprotein (N), envelope (E) protein, and membrane (M) protein (Figure S1). The frequency of mutations on S protein is higher than other parts in different SARS-CoV-2 variants.

The S protein of SARS-CoV-2 consists of 1273 amino acids containing subunits S1 and S2 (Figure S2A). The subunit S1 is divided into N-terminal domain (NTD), C-terminal domain (CTD), and receptor-binding domain (RBD) that binds with angiotensin-converting enzyme 2 (ACE2). To analyze the similarities and mutations of S protein in different SARS-CoV-2 variants, we aligned the S proteins' sequences in eleven SARS-CoV-2 strains, including original, D614G, Alpha, Beta, Gamma, Delta, Kappa, Lambda, Gamma, Iota, and 21H variants. The results showed that the S proteins' sequences have a mean homology of 99.51%, and the common amino acid mutations among these SARS-CoV-2 strains are mainly enriched at S1 NTD and RBD (Figure S2B). The D614G mutation is most common and occurs in ten SARS-CoV-2 variants (Figure S2B), while the E484K and N501Y mutations appear 4 times.

### 2.2. The Spike Protein Structure Prediction of Ten SARS-CoV-2 Strains

**Figure 1:**
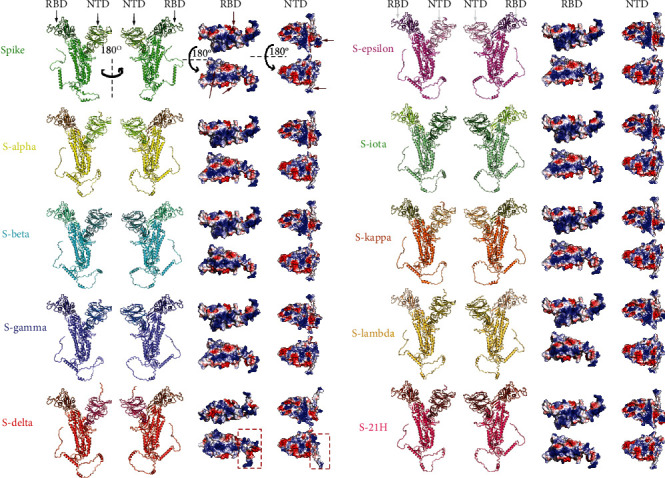
Spike structure prediction of ten SARS-CoV-2 strains. The structures of full length, receptor-binding domain (RBD), and S1 N-terminal domain (NTD) of spike protein were shown, respectively. Monomer spike proteins were displayed in the cartoon model. RBD and NTD were shown in the electrostatic surface. Red and blue indicate negative and positive charges, respectively. Red arrows indicate the significantly changed sites on RBD and NTD among ten strains. Red boxes indicate the major different parts of RBD and NTD in the Delta variant compared with the original spike protein.

The alterations of S protein in different SARS-CoV-2 variants have been reported to affect virulence, transmissibility, disease severity, and immune escape [12, 13, 18]. However, the influence of amino acid mutations on S protein structure is not yet clear. To acquire the spike structures of different SARS-CoV-2 strains, we used AlphaFold to predict their spike protein structures based on the mutated protein sequences. The spike proteins of ten major worldwide spread SARS-CoV-2 strains were successfully predicted, including original, alpha, beta, gamma, delta, epsilon, iota, kappa, lambda, and 21H strains. The full-length spike protein monomers are presented, and the RBD and NTD of the S1 protein are marked by different colors (Figure 1). To compare the structural difference among the ten SARS-CoV-2 strains, we performed the pairwise structural alignment based on full-length S (Figure S2), NTD (Figure S3), and RBD (Figure S4), respectively. The comparison of the full-length spike protein structures shows that the parts of S1 RBD and NTD are significantly changed whereas the S1 C-terminal domain and S2 domain are hardly affected (Figure S3). The comparison of S1 NTD and RBD structures predicted based on their sequences shows that NTD structures are diverse in different strains, and especially the N-terminal structures of NTD are significantly changed (Figure S4). The N-terminal and receptor-binding motif (RBM) in RBD structure domains display significant changes (Figure S5). These observations indicate that S1 RBD and NTD of spike proteins have significant structural changes between different SARS-CoV-2 strains. We further compared the electrical property on the structures of RBD and S1 NTD of ten major SARS-CoV-2 strains. The results showed that amino acid mutations could change the electrical property on the RBD and S1 NTD surfaces (Figure 1). Particularly, compared with the original S protein, RBD and S1 NTD of SARS-CoV-2 Delta variants were notably different structures and electrostatic surfaces (Figure 1 red box and S3-S5).

### 2.3. Validation of the Predicted S Protein Structures of SARS-CoV-2

**Figure 2:**
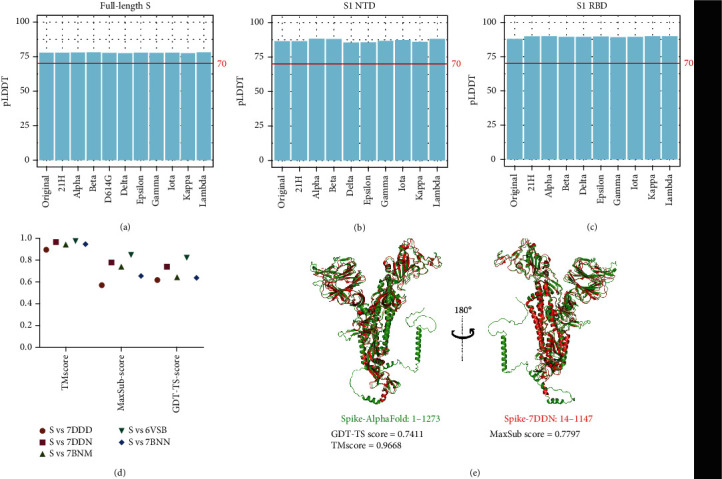
Validation of the predicted S protein structures. (a–c) The mean pLDDT values of full-length S proteins (a), S1 NTD (b), and S1 RBD (c) in different SARS-CoV-2 variants. (d) Comparison of spike protein between AlphaFold predicted and experimental structures. The experimental structures were downloaded from the protein data bank (PDB), and their accession numbers were labeled. (e) Alignment of AlphaFold prediction and experimental structure (PDB: 7DDN). The prediction and experimental structures are colored in green and red, respectively.

To evaluate the predicted S protein structures of SARS-CoV-2 variants, we analyzed the values of the predicted Local Distance Difference Test (pLDDT) calculated by AlphaFold since this value represents the domain accuracy [36]. The pLDDT value above 70 indicates the structures have been considered as confidently predicted structures [37]. Our results show that mean pLDDT values of full-length S, S1 NTD, and S1 RBD in different SARS-CoV-2 strains are all above 75 (Figures 2(a)–2(c)). We also found that percentages of amino acid residues whose pLDDT values are above 70 in all residues of different full-length S are all higher than 76%, and the percentages of different S1 NTD, and S1 RBD are above 82%, and 89%, respectively (Figure S6). These results of pLDDT indicate the predicted structures are highly accurate.

Furthermore, we compared the AlphaFold-predicted S protein structure of the original strain with five experimental S proteins, including PDB ID: 7DDD, 7DDN, 7BNM, 6VSB, and 7BNN to evaluate the validity of the predicted structures. We calculated Template Modelling (TM) score [38], maximal subset (MaxSub) score [39], and Global Distance Test (GDT)-TS score [40] between the AlphaFold-predicted and experimental S proteins (Figure 2(a)). The resulting TM scores were >0.88, the MaxSub scores > 0.5, and the GDT − TS score > 0.6 (Figure 2(d)), which indicated the predicated S proteins were of high confidence. We further analyzed the 3D structure similarities between the predicated original S protein with 7DDN that is at an open state of S protein. The aligned structure showed the predicated original S protein and 7DDN were highly similar (Figure 2(e)). We find that the S1 NTD and RBD structures are also similar to the experimental structure of 7DDN. Overall, these results validate the high accuracy and reliability of AlphaFold at predicting the S protein of SARS-CoV-2.

### 2.4. Classification of SARS-CoV-2 Strains Based on the Genomic Sequences and Protein Structures

**Figure 3:**
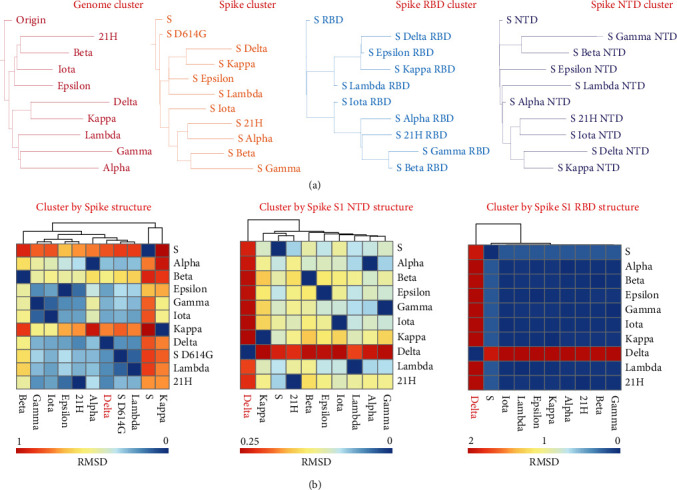
Cluster analysis of SARS-CoV-2 strains based on protein sequences and structures. (a) Four kinds of clusters of SARS-CoV-2 strains are based on protein sequences. The genome cluster is based on the genome sequences. The spike, spike RBD, and spike NTD clusters are based on their protein sequences. (b) Three kinds of clusters of SARS-CoV-2 strains are based on protein structures. Structural similarities are evaluated by RMSD related to Table S1.

Successful classification of SARS-CoV-2 strains is important to explore the development of the virus and predict its evolution. To classify the SARS-CoV-2 strains, we performed phylogenetic analyses based on the genomic and protein sequences of all strains. The results showed that SARS-CoV-2 Delta and kappa variants are highly homologous in four sequence-based clusters (Figure 3(a)). Clusters based on the spike protein, S1 RBD, and S1 NTD showed SARS-CoV-2 gamma and beta variants are highly homologous (Figure 3(a)). The cluster trees of spike protein RBD and full-length spike proteins are highly similar, which indicates the spike variances could be mainly from mutations on the spike RBD domains. Next, we further performed clustering analyses based on the similarities of the full-length spike protein, S1 NTD, and S1 RBD structures. Notably, clusters based on S1 NTD and RBD revealed that the SARS-CoV-2 Delta variant is significantly different from other variants (Figure 3(b) and Table S1). The root means square deviation (RMSD) values of S1 RBD and NTD on the SARS-CoV-2 Delta variant are higher than that of other variants, indicating that the S1 and RBD structure of the Delta variant has changed significantly more when compared with the other strains. Overall, the clusters based on structures and sequences of full-length spike protein are different, and the sequence could not well reflect the structures of these SARS-CoV-2. These results suggest that the structure of Delta S1 RBD and NTD are unique compared to other variants. Currently, the SARS-CoV-2 Delta variant is spreading quickly across the world and possesses high virulence and resistance to available vaccines. Thus, the unique structures of SARS-CoV-2 Delta S1 RBD and NTD could play important roles in increasing the detrimental change in COVID-19 epidemiology.

### 2.5. The Comparison of S1 NTD Structure between SARS-CoV-2 Delta and Original Strains

**Figure 4:**
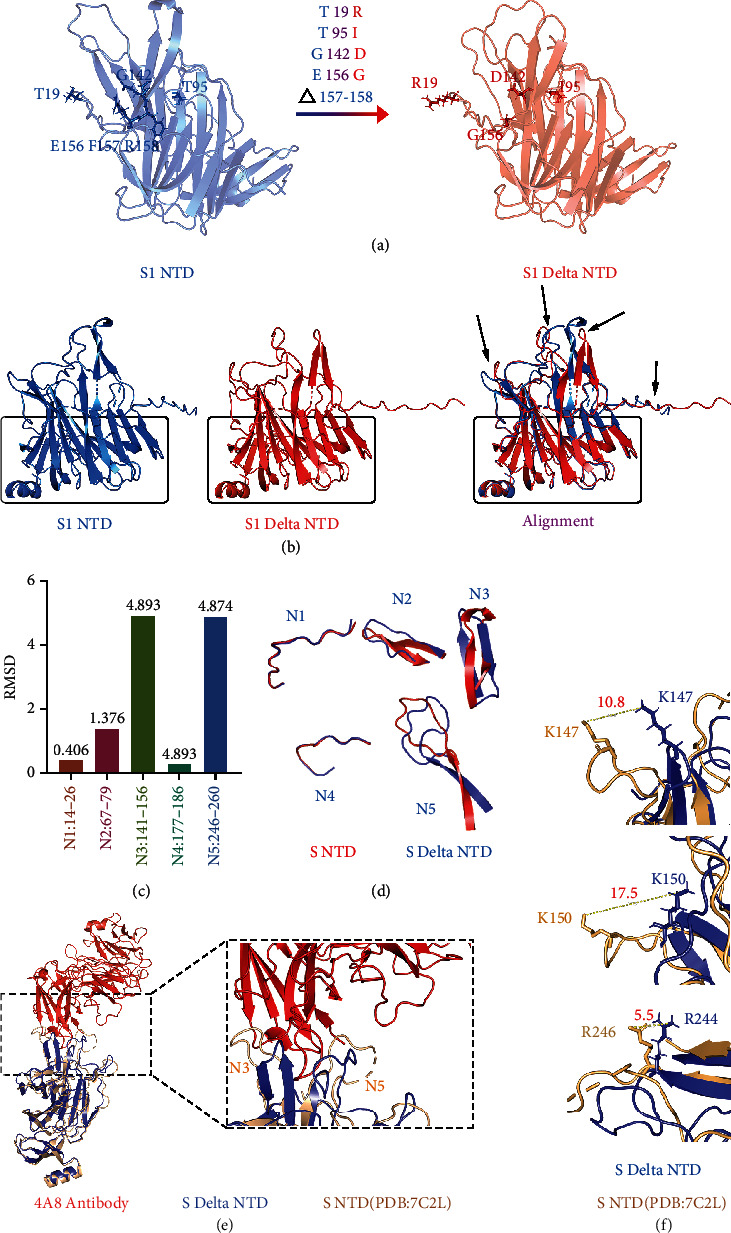
Comparison of spike protein substrate 1 NTD between SARS-CoV-2 Delta and original strains. (a) Structural changes of Delta strain S1 NTD compared to the original strain. Black arrows and boxes indicate the structures with and without changes, respectively. (b) Mutations of amino acids on Delta S1 NTD. Triangle represents deletion of amino acid. (c, d) Comparison of five loops of S1 NTD. The RMSD values are shown in (c). The structural comparisons are shown in (d). (e) The interaction between NTD and 4A8 antibody (PDB: 7C2L). (f) The differences of three sites on loop N3 and N5 between Delta and original NTD. Yellow lines indicate the distance between corresponding amino acids.

To explore the structural difference of S1 NTD between Delta and original (wild-type) strains, we analyzed the effects of amino acid (AA) mutations on the S1 NTD structure of the Delta strain. The result shows that six amino acids are mutated on the S1 NTD structure of Delta strain compared with the original strain, including T19R, T95I, G142D, E156G, and deletions of F157 and R158 (Figure 4(a)). These mutations have changed the molecular orientation and electrical properties of the protein, such as uncharged T19 is mutated to positively charged R19, aliphatic G142 is mutated to negatively charged D142, and negatively charged E156 is mutated to aliphatic G156 (Figures 1 and 4(a)). Meanwhile, we observed the effects of these AA mutations on S1 NTD structures. The results showed that the domains enriched AA mutations are significantly changed compared with the original strain (Figure 4(b) black arrows), while the no mutated domains of Delta S1 NTD have high similarities with the original strain (Figure 4(b) black box).

The S1 NTD has been confirmed as an epitope that could be bound with several antibodies [34, 41]. To further investigate the effects of structural changes on binding antibodies, we calculated the RMSD values of five loops of S1 NTD between Delta and original strains, including N1 loop (residues 14-26), N2 loop (residues 67-79), N3 loop (residues 141-156), N4 loop (residues 177-186), and N5 loop (residues 246-260). The results show that RMSD values of N3 and N5 are greater than 4.8, and RMSD values of N1, N2, and N4 are 0.406, 1.376, and 0.273, respectively (Figure 4(c)). The comparison of structures also shows N3 and N5 are significantly different (Figure 4(d)). Given that the N3 and N5 loops mediate the interaction with antibody [41], we observed the interaction between S1 NTD and antibody 4A8 (PDB: 7C2L). Compared with the original strain, N3 and N5 of Delta S1 NTD are significantly different, and the pivotal hydrophilic interaction domain constructed with Delta NTD loops of N3 and N5 and three complementarity-determining regions (CDRs) are much more open than that of the original strain (Figure 4(e)).

Meanwhile, skewings of K147, K150, and R246 responsible for forming salt bridges and hydrogen (H)–bonded with 4A8 between Delta and original NTD are 10.8, 17.5, and 5.5 angstroms, respectively (Figure 4(f)). We evaluated the interactions between NTD and antibody 4A8 by HADDOCK and HDOCK prediction. The HADDOCK score, van der Waals energy, desolvation energy, and RMSD from the overall lowest-energy structure of the original-NTD-4A8 complexes are much lower than that of the Delta-NTD-4A8 complexes, while the electrostatic energy, *Z*-score, and HDOCK docking score of the complexes are hardly influenced (Table 1 and S2). These results indicate that AA mutations on Delta S1 NTD significantly affect the structure and alter the interactions between NTD and antibodies.

### 2.6. The Comparison of S1 RBD Structure between SARS-CoV-2 Delta and Original Strains

**Figure 5:**
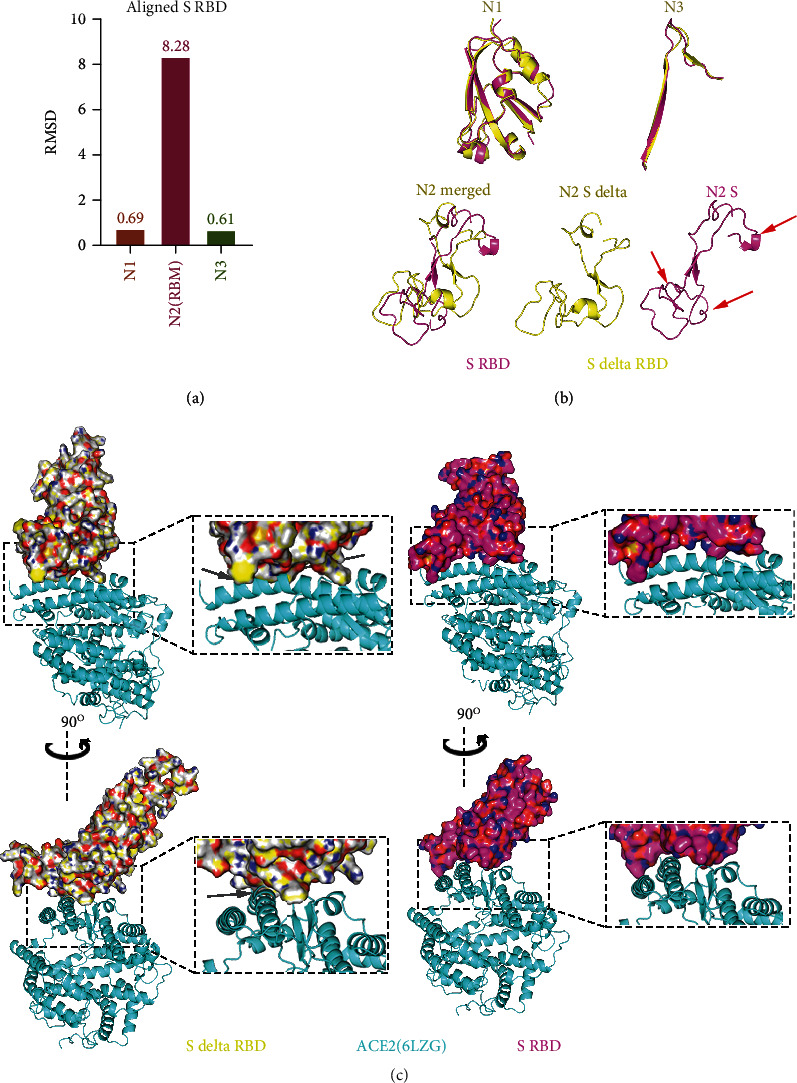
Comparison of spike protein RBD between SARS-CoV-2 Delta and original strains. (a, b) Comparison of three loops of spike RBD. The RMSD values are shown in (a). The structural comparisons are shown in panel (b). Red arrows indicate the structures with significant changes. (c) Interactions between spike RBD and ACE2. Spike RBD and ACE2 are downloaded from PDB (6LZG).

In addition to AA mutations on S1 NTD, two AA mutations-L452R and T478K occur on the Delta S1 RBD domain, which could change AA's electrical properties; the aliphatic L452 and polar uncharged T478 are mutated to positively charged R452 and K478, respectively (Figure 1). To explore the structural difference of S1 RBD between Delta and original strains, we compared the S1 RBD domains. The S1 RBD could be divided into three parts, including N1 (residues 333 to 438), receptor-binding motif (RBM, residues 438 to 506), and N3 (residues 507 to 539), based on the structure. The RMSD values of N1, RBM, and N3 between Delta and original strains are 0.69, 8.28, and 0.61, respectively (Figure 5(a)). Consistently, the structural comparisons show that RBM domains are significantly different between Delta and original strains, while domains of N1 and N3 are highly similar (Figure 5(b)). Given that the RBM domain plays an important role in binding with ACE2, we explored the interactions between ACE2 and Delta S1 RBD domain by aligning structures based on their homologous sequence. Compared with the original strain, Delta S1 RBD displays a close interaction with ACE2 (Figure 5(c)).

Moreover, we performed docking simulations to quantify the interactions between ACE2 and RBD structures of all mutants. The results show that AA mutations could affect the docking scores between ACE2 and RBD in all SARS-CoV-2 mutants, indicating that interactions between ACE2 and RBD are changed (Table 2 and S3). Comparison between original and Delta strains shows that electrostatic energy, desolvation energy, *Z*-score, and RMSD from the overall lowest-energy structure are low while van der Waals energy and HADDOCK score are high in Delta-RBD-ACE2 complexes (Table 2). Most notably, significant differences in electrostatic energy between the original and Delta complexes are observed. The electrostatic energy of the original strain is −221.5 ± 11.0, whereas that of the Delta variant is −264.5 ± 23.9 (Table 2), which suggests that the mutated positively charged R452 and K478 on Delta RBD decrease electrostatic energy. Overall, these results indicate that AA mutations on the Delta RBD domain could significantly alter the RBD structure and its interactions with ACE2.

### 2.7. The Effects of AA Mutations on Interactions between S1 RBD and Antibodies

**Figure 6:**
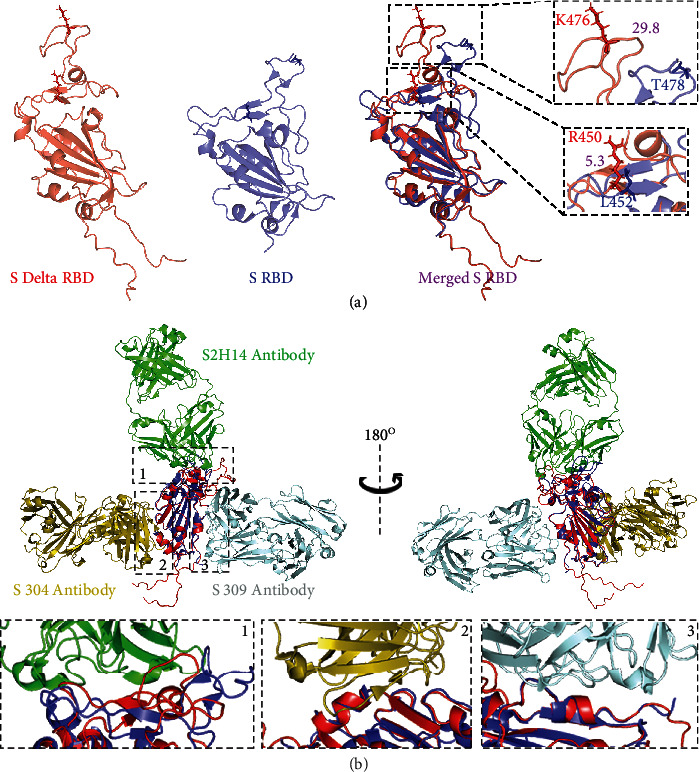
The effects of Delta strain mutations on interactions between RBD and antibodies. (a) Comparison of two mutated amino acids on RBD. (b) The interactions between RBD and antibodies. Antibodies and original RBD are downloaded from PDB (7JX3).

The structural comparison of S1 RBD between Delta and original strains shows that mutations on T478 and L452 induce significant skewing of AAs and alteration of the structures (Figure 6(a)). The structural difference occurs on RBM where AA mutations are enriched (Figure 6(a)), which is consistent with the results of RMSD values (Figure 5(a)). Distances between T478 and L452 of the original and the corresponding K476 and R450 of the Delta are 29.8 and 5.3 angstrom, respectively (Figure 6(a)). Given that three epitopes of S1 RBD could be recognized and bound by antibodies, we analyzed interactions between S1 RBD and antibodies. The results show that the structures of two of three epitopes are slightly changed, and the bindings with S304 and S309 antibodies are almost not influenced (Figure 6B2 and B3), whereas the epitope bound with S2H14 antibody, also involved in binding with ACE2, is significantly changed indicating that the capacity of this epitope bound with antibodies is decreased. To evaluate the interactions between the antibody S2H14 and S1 RBD of original and Delta strains, we performed docking simulations based on the interactions between the heavy chain of the S2H14 antibody and S1 RBD. The results showed that the original-RBD-S2H14 complex has a higher binding affinity than the Delta-RBD-S2H14 complex (Table 3 and S4). The values of the HADDOCK score, electrostatic energy, desolvation energy, and RMSD from the overall lowest-energy structure in the original-RBD-S2H14 complex are much lower than those in the Delta-RBD-S2H14 complex, while Van der Waals energy in the original-RBD-S2H14 complex is higher than that in the Delta-RBD-S2H14 complex (Table 3). These results indicated that the structural changes of Delta S1 RBD induced by amino acid mutations altered the epitope and reduced antibody recognition.

### 2.8. Virtual Screening of Potential Drugs Targeting Delta S1 NTD

**Figure 7:**
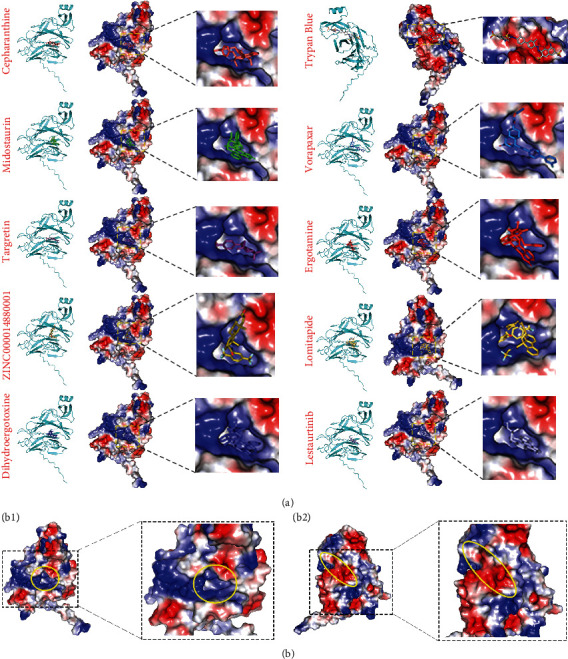
Complexes of top 10 drugs targeting Delta S1 NTD domain. (a) Structures of top ten drugs combined with Delta NTD. (B1 and B2) Two major pharmacophores on Delta NTD. Red and blue colors on the surface indicate negative and positive charges, respectively. Yellow circles indicate the pharmacophores. Drugs used for virtual screening are downloaded from the ZINC database and filtered by “world subset.” The values are related to table S5.

**Figure 8:**
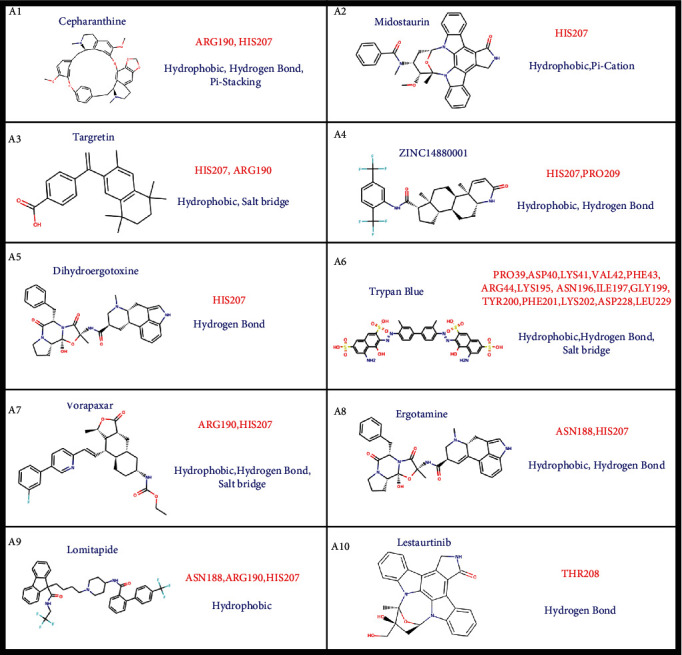
Chemical structures of top 10 drugs targeting Delta S1 NTD. The drug interacts with the red amino acids on the Delta S1 NTD domain based on the blue interactions, including hydrophobic interaction, hydrogen bond, pi-stacking, pi-cation, and salt bridge related to Figure 7 and Table S5.

The high accurate structural prediction of SARS-CoV-2 spike protein based on AlphaFold provides us with credible models for screening potential compounds targeting these proteins. Given that the SARS-CoV-2 Delta variant has been a major threat worldwide, we performed the virtual screening technique to identify potential drugs. To identify chemicals that can be applied in the clinic, we selected a database consisting of 5903 approved drugs worldwide (Table S5). We utilized the screening approach to calculate the binding affinity of the compounds targeting Delta S1 NTD distinct from other variants. The results revealed 40 kinds of drugs targeting S1 NTD that display high binding affinity, whose binding energies are less than -9 kcal/mol (Table S5). The top 10 best docking drugs targeting S1 NTD are cepharanthine, midostaurin, targretin, zinc000014880001, dihydroergotoxine, trypan blue, vorapaxar, ergotamine, lomitapide, and lestaurtinib (Figure 7(a) and Table 4). These 10 drugs are enriched at two pharmacophores on the Delta NTD (Figure 7(b)). Pharmacophore 1 is a cavity displaying a positive charge, while pharmacophore 2 shows a negative charge (Figure 7(b)). Trypan blue among the ten drugs is targeted pharmacophore 2, and the other nine drugs are targeting pharmacophore 1 (Figure 7). The main interactions between trypan blue and pharmacophore 2 are hydrophobic, hydrogen bonds, and salt bridges through binding with PRO39, ASP40, LYS41, VAL42, PHE43, ARG44, LYS195, ASN196, ILE197, GLY199, TYR200, PHE201, LYS202, ASP228, and LEU229 (Figure 8). The nine drugs interact with ASN188, ARG190, HIS207, THR208, and PRO209 on the pharmacophore 1 through hydrophobic interactions, hydrogen bonds, pi-cation, and salt bridge (Figure 8).

### 2.9. Virtual Screening of Potential Drugs Targeting Delta S1 RBD and RBM

**Figure 9:**
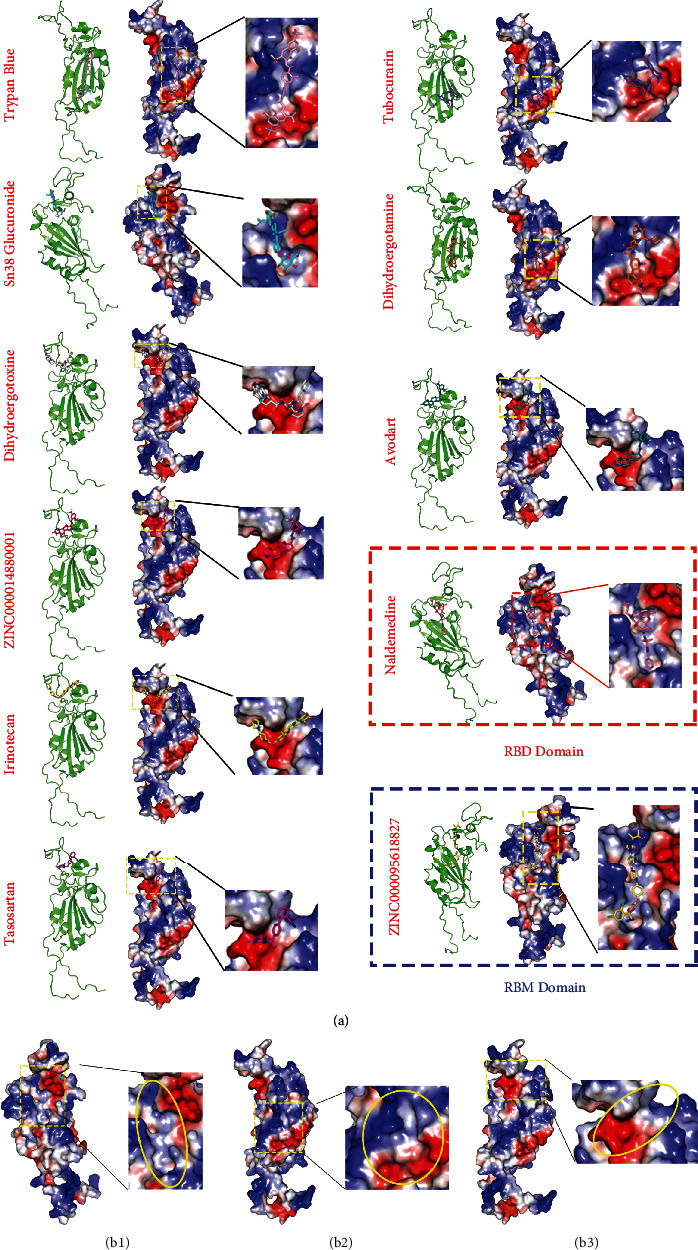
Complexes of top 10 drugs targeting Delta S1 RBD domain. (a) Structures of top ten drugs combined with Delta S1 RBD and RBM. The top nine drugs are commonly identified in S1 RBD and RBM. Red and blue boxes indicate the unique drugs screened in S1 RBD and RBM, respectively. (B1–3) Three major pharmacophores on Delta S1 RBD and RBM. Red and blue colors on the surface indicate negative and positive charges, respectively. Yellow circles indicate the pharmacal cavities. Drugs used for virtual screening are the same as Figure 7.

**Figure 10:**
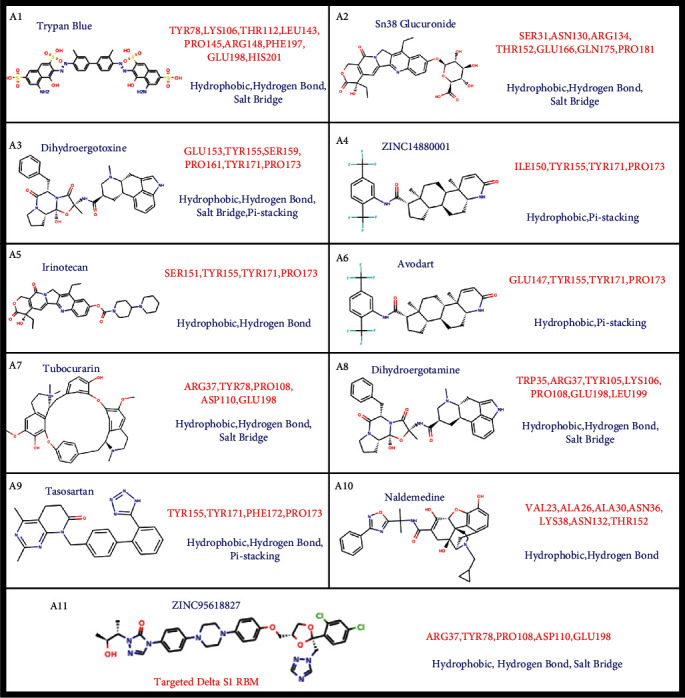
Chemical structures of drugs targeting Delta S1 RBD and RBM. Chemicals A1 to A9 are common drugs in S1 RBD and RBM. Chemicals A10 and A11 are unique for RBD and RBM, respectively. The drug interacts with the red amino acids on the Delta S1 NTD domain based on the blue interactions, including hydrophobic interaction, hydrogen bond, pi-stacking, pi-cation, and salt bridge related to Figure 9 and Table S5.

Given that the Delta S1 RBD and RBM play important roles in entering the host cells through binding with ACE2, Delta S1 RBD, and RBM have great potential as new drug targets. To further search potential drugs targeting the SARS-CoV-2 Delta strain, we performed the virtual screening methods on the Delta S1 RBD and RBM, with RBM used for cross-validation. The results showed that 14 and 16 kinds of drugs showed a high binding affinity with the Delta S1 RBD and RBM, respectively (Table S5). Nine of the top 10 best docking drugs are significantly overlapped in targeting S1 RBD and RBM while the binding energies are different, which include trypan blue, sn38 glucuronide, dihydroergotoxine, zinc000014880001, irinotecan, avodart, tubocurarin, dihydroergotamine, and tasosartan (Figures 9(a) and 10 and Table 4). ZINC95618827 uniquely interacts with ARG37, TYR78, PRO108, and GLU198 on Delta S1 RBM by hydrophobic interactions, hydrogen bonds, and salt bridge (Figure 10A11). Naldemedine interacts with VAL23, ALA26, ALA30, ASN36, LYS38, ASN132, and THR152 on the Delta S1 RBM by hydrophobic interactions and hydrogen bonds (Figure 10A10). The identified drugs are enriched at three pharmacophores on Delta RBD (Figure 9(b)). Sn38 glucuronide, naldemedine, and zinc95618827 are enriched at pharmacophore 1 (Figure 9 B1), while trypan blue, tubocurarin, and dihydroergotamine are enriched at pharmacophore 2 forming hydrophobic interactions, hydrogen bonds, and salt bridge (Figures 9B2 and 10). Dihydroergotoxine, zinc000014880001, irinotecan, tasosartan, and avodart are enriched at pharmacophore 3 forming hydrophobic interactions, hydrogen bonds, salt bridge, and pi-stacking (Figures 9B3 and 10).

**Figure 11:**
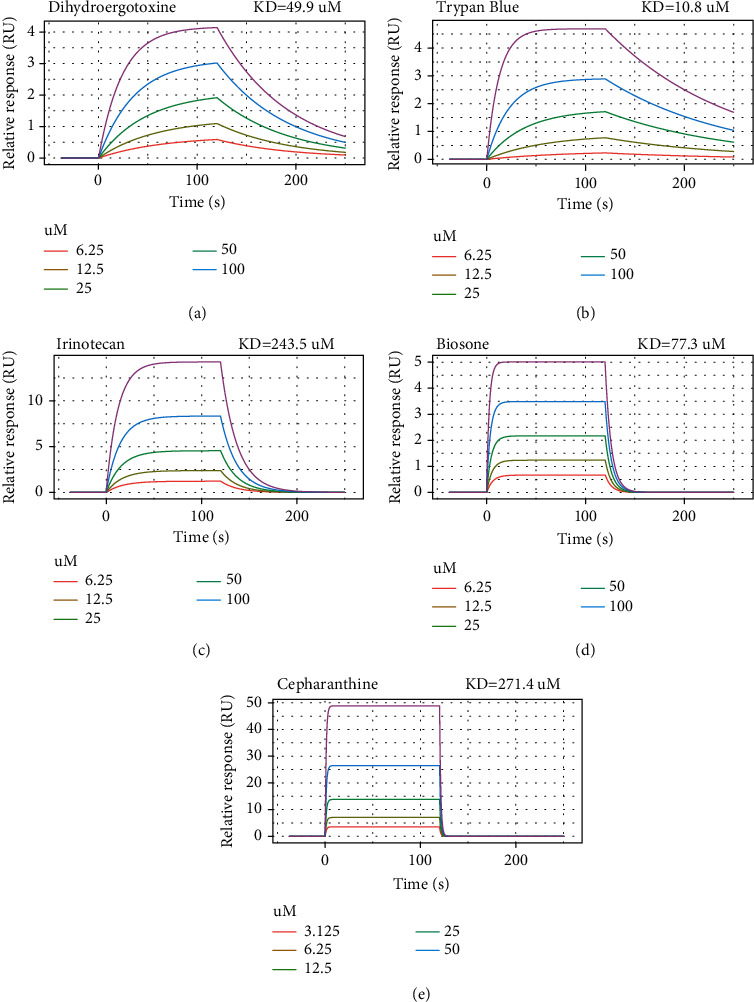
The five compounds exhibit high binding affinity to S1 RBD of the Delta variant. The estimated KD constants of dihydroergotoxine (a), trypan blue (b), irinotecan (c), biosone (d), and cepharanthine (e) are 49.9, 10.8, 243.5, 77.3, and 271.4 *μ*M, respectively.

To validate the binding affinity of the screened compounds to Delta S1 RBD, we used surface plasmon resonance technology (BIAcore 8K, GE Healthcare) to analyze the binding energy which is a frequently used instrument to detect the binding energy between proteins and small molecule compounds [42, 43]. We detected the binding energy between S1 RBD of Delta variant and the 8 screened compounds, including dihydroergotoxine, trypan blue, irinotecan, biosone, cepharanthine, avodart, tasosartan, and conivaptan. Five of the compounds exhibited high binding affinity to S1 RBD of the Delta variant, and the estimated binding affinity values (KD) of dihydroergotoxine, trypan blue, irinotecan, biosone, and cepharanthine were 49.9, 10.8, 243.5, 77.3, and 271.4 *μ*M, respectively (Figure 11). The results indicated that these compounds could have antiviral activity and that the drugs predicted by bioinformatics methods were highly confident.

## 3. Discussion

AlphaFold v1 has been used to predict the structures of many SARS-CoV-2 proteins, such as SARS-CoV-2 ORF 6, 8, 10 and NSP 2, 3, 4, 6 [44, 45], which with high accuracy. In this study, we focused on predicting the S protein structure of ten SARS-CoV-2 strains, including original, Alpha, Beta, Gamma, Delta, Kappa, Lambda, Gamma, Iota, and 21H variants with high accuracy by applying the machine learning method, AlphaFold [37]. The reliability of the predicted S protein structures was validated in two ways, firstly by use of the pLDDT value calculated by AlphaFold and the comparison between AlphaFold-predicted and experimental S protein structures (Figure 2). To the best of our knowledge, this is the first study to present the S protein structures of so many SARS-CoV-2 strains, which helps lay a foundation for further SARS-COV-2 research and supplement experimental structural biology. Moreover, this effective utilization of AlphaFold may help the developers to update and optimize the algorithm.

How to correctly classify the different SARS-CoV-2 strains is an important question; the main method used to classify the SARS-CoV-2 strains so far has been phylogenetic analysis, which is based on the genomic sequence [12, 15, 27, 46, 47]. While phylogenetic analyses do a good job of reflecting the evolutionary trajectory of the virus to some extent, it cannot account for differences in virulence of the strains and is not able to substantially differentiate between the Delta and other strains. Trying to solve this problem, we attempted to classify the SARS-CoV-2 strains by using structural similarity. Interestingly, our classifications showed the unique cluster of the Delta strain based on the structural similarity of both S1 NTD and RBD, which was different from that based on the sequences (Figure 3). This result can be interpreted as that the structural difference can reflect the functional variation more directly. This study suggests that structural similarity can be a new way to classify SARS-CoV-2 strains under the novel conditions that AlphaFold can provide high accuracy protein structures.

SARS-CoV-2 Delta (B.1.617.2) strain was first identified in India in December 2020, which has become a major epidemic strain worldwide accounting for more than 80% of new cases (nextstrain/ncov) [47]. The SARS-CoV-2 Delta strain has been confirmed to be less sensitive to serum neutralizing antibodies and vaccine-elicited antibodies, compared with wild-type strain [48, 49]. Here, we predicted the high-accuracy spike protein structure of SARS-CoV-2 Delta strain with powerful tool-AlphaFold and demonstrated that the structures of S1 NTD and RBD of SARS-CoV-2 Delta strain were significantly changed. They displayed lower binding affinities with the corresponding antibodies, 4A8 and S2H14 antibodies, compared with the original stain (Figures 4 and 6). The lower binding affinities with antibodies can reduce the neutralization ability to the spike protein of SARS-CoV-2 Delta strain and then induce immune evasion. Our results provided a bioinformatic and computational insight into explaining how the SARS-CoV-2 Delta variant causes immune evasion from spike protein structure, and it could provide a reference for the follow-up research. These methods presented in our research can also be used to continuously monitor the mutation and structural variation of SARS-CoV-2 strains.

Effective drugs against SARS-CoV-2, especially the Delta strain, have not yet been developed. Virtual screening combined with structural biology has been a useful assistant method for drug discovery [50–52]. The high-accuracy predicted structures of spike proteins provide us a basis for the virtual screening. The S1 NTD and RBD are crucial parts for binding host cells [53, 54], and thus, the binding of the drug with these parts could play important roles in inhibiting the infection of SARS-CoV-2. Given the unique structure and pandemic of the Delta strain, we selected 5903 worldwide approved drugs that can be used in the clinic rapidly from the ZINC database (https://zinc15.docking.org/) for virtual screening of targeting S1 NTD and RBD of Delta strain. After a strict filter at binding energy less than -9 kcal/mol, 40 and 14 kinds of drugs targeting S1 NTD and RBD were identified. These drugs can form hydrophobic interactions, hydrogen bonds, salt bridges, Pi-cation, and Pi-stacking with key amino acids on S1 NTD and RBD. Some of these drugs, including Dihydroergotoxine, Avodart, Tubocurarine, and Irinotecan, have been evaluated to potentially affect SARS-CoV-2 towards proteases, and NSP9 [55–58]. Our results indicated that these drugs individually or in combinations may be used as potential inhibitors of S1 NTD and RBD of SARS-CoV-2 after verifying their in vivo and in vitro antiviral ability.

A limitation of this study is that we only focused on the S protein structures of SARS-CoV-2. Besides the S protein, other parts, including nucleocapsid protein, envelope protein, and RNA-dependent RNA polymerase of SARS-CoV-2, are also not well known, while they can play important roles in SARS-CoV-2 replication and infection. These proteins should be explored in future works following our methods. Moreover, the availability of screened drugs targeting the Delta strain S1 NTD and RBD should be verified by the strict experiments before applying to the clinic. We would like to highlight that the current study is mainly based on computation and simulation providing essential data; however, some structures and results should be evaluated by further experiments.

In conclusion, our study acquired the high-accuracy spike protein structures of ten SARS-CoV-2 variants by using the AlphaFold model. The complementary methods, containing the comparison with experimental structures and the validation of pLDDT of the AlphaFold built-in algorithm, verified the structures were highly accurate. We found that the clustering analysis based on structural similarity could reflect the current characteristics of the epidemic more accurately than those based on the protein sequence, such as the mainly pandemic SARS-CoV-2 Delta variants displayed the unique features in the cluster based on the structural similarity. Moreover, the analysis of the binding affinities of ACE2-RBD, antibody-NTD, and antibody-RBD complexes in the different variants revealed that the recognition of antibodies against S1 NTD and RBD was decreased in the variants, especially the Delta variant compared with the original strain, which may induce the immune evasion of SARS-CoV-2 variants. Furthermore, we identified multiple compounds that target S1 NTD and RBD by virtual screening the ZINC database against a high-accuracy predicted structure of Delta spike protein, which might contribute towards the development of clinical anti-SARS-CoV-2 medicines. Our study provided abundant basic data for further research related to curing COVID-19.

## 4. Material and Methods

### 4.1. Structure Modeling with AlphaFold

The structural prediction of spike protein was based on the model of AlphaFold v2.0 entered in CASP14 and published in Nature [37]. The inference pipeline and source code of AlphaFold are available under an open-source license at https://github.com/deepmind/alphafold. The parameter of preset is the same as that used in CASP14, which runs with all genetic databases and with 8 ensembles. The mirrored databases used in our study include BFD [59], MGnify clusters [60], UniRef90 [61], Uniclust30 [62], protein data bank (PDB), and PDB70 [63]. The spike protein sequences of different SARS-CoV-2 variants downloaded from GISAID (http://gisaid.org) were used as inputs. The graphics processing unit (GPU) used in this study was NVIDIA Tesla V100 on the *π* 2.0 cluster supported by the Center for High-Performance Computing at Shanghai Jiao Tong University.

### 4.2. Calculation of Similarity between Experimental and Predicted Structures

The experimental structures of spike protein were downloaded from PDB, including 7DDD, 7DDN, 7BNM, 6VSB, and 7BNN. The 3D protein structures were visualized by the software PyMol. The similarity between experimental and predicted structures was evaluated by template modelling (TM) score, maximal subset (MaxSub) score, global distance test (GDT)-TS score, root-mean-square deviation (RMSD), and the predicted local-distance difference test (pLDDT). TM-score [38], MaxSub score [39], and GDT-TS score [40] were calculated based on the online service of TM-align (https://zhanggroup.org/) [64, 65]. The RMSD and pLDDT between experimental and predicted structures were calculated by the software PyMol and AlphaFold, respectively [36, 66].

### 4.3. Cluster Analyses of Different SARS-CoV-2 Strains

The cluster analyses were performed based on both protein sequences and structures. The software Molecular Evolutionary Genetics Analysis-X (MEGA-X) was used for phylogenetic analyses based on protein sequences. The ClustalW alignment algorithms were used to align the multiple sequences, and evolutionary tree was generated using the maximum likelihood method [67]. The structural cluster analyses were based on the matrix of RMSD values between different SARS-CoV-2 strains, and the method used to cluster these strains was based on the built-in algorithm of R packages “pheatmap.” The data were visualized by the R packages of “pheatmap” and “ggplot2.”

### 4.4. Consensus Docking of S1 NTD and Antibody 4A8

The relaxed-predicted S1 NTD structures of different SARS-CoV-2 strains were selected for the modeling of biomolecular complexes between S1 NTD and antibody 4A8 [41] based on high ambiguity-driven protein-protein docking (HADDOCK) [68, 69]. The interface residues at 25 to 32, 51 to 58, 100 to 116 for antibody 4A8 M chain, and at 145 to 150 for S1 NTD were determined for restraint docking in an online HADDOCK server. The other docking parameters were set as default. The algorithm of HDOCK combined both template-based and free approaches were deployed for cross-validation [70, 71].

### 4.5. Consensus Docking of S1 RBD with ACE2 and Antibodies

Similarly, the docking analyses of ACE2 and antibodies with S1 RBD have followed the same method as described in Section 2.4. Briefly, for antibody S2H14 [9] docking against the S1 RBD, the interface residues at 52, 56, 107, and 108 on the heavy chain of the antibody S2H14, and 449, 498, 500, and 505 (131,180,182, and 187) on S1 RBD were determined for restraint docking. For ACE2 docking against S1 RBD, the interface residues at 21, 24, 27, 28, 30, 35, 38, 79, 80, 82, 83, and 353 for ACE2 and at 449, 453, 455, 456, 486, 487, 489, 493, 496, 498,500, 501, 502, and 505 (131, 135, 137, 138, 168, 169, 171, 175, 178, 180, 182, 183, 184, and 187) for S1 RBD were determined for restraint docking. The online HADDOCK server was used for docking analysis, and the HDOCK was used for cross-validation.

### 4.6. Virtual Screening of Potential Drugs Targeting S1 NTD and RBD

For fast use of the screened drugs in the clinic, the 5903 chemicals used for virtual screening were downloaded from the ZINC database under the filter of the world-approved drugs. The software of Autodock vina [72] was applied to identify potential drugs binding with S1 NTD and RBD. The S1 RBD and NTD were included in the searching grid boxes, respectively. The binding sites and pharmacophores were searched automatically by the software. Moreover, for cross-validation, the S1 RBM was set as a target in the searching grid box. Finally, based on the docking scores, the screened compounds with the threshold values of binding energy fixed less than -9.00 kcal/mol were identified as potential drugs for S1 NTD and RBD. The binding affinity was measured by surface plasmon resonance technology using a BIAcore 8K instrument (GE Healthcare, United States) with running buffer (PBST containing 5% DMSO) at 25°C. The S1 RBD of Delta (Beyotime Biotechnology Co., LTD, China, #P2341) was purchased from immobilized onto sensor CM5 chips by a standard amine-coupling procedure in 10 mM sodium acetate (pH 5.5). Compounds were serially diluted and injected into the chip at a flow rate of 30 *μ*l/min for 120 s (contact phase), followed by 120 s of buffer flow (dissociation phase). The binding affinity of KD value was calculated by the software of Biacore Insight Evaluation V3.0 (GE Healthcare, United States).

### 4.7. Interaction Analysis between Potential Drugs and Protein Structures

The top ten screened compounds ranked by the lowest binding energy were selected for interaction analysis with S1 NTD and RBD. The interaction analysis was performed using the online service of protein-ligand interaction profiler (PLIP) [73]. The algorithm of PLIP identified the amino acids on protein structures responsible for forming specific interactions with the chemicals. The 3D structures of compounds, S1 NTD, and RBD were visualized by the software PyMol, and the combination compounds with S1 NTD and RBD were generated by the software Autodocktools.

### 4.8. Statistical Analysis and Data Visualization

The statistical analysis was performed using R software (Version 4.0.3) on Rstudio and Microsoft Excel (Version 2019). The data visualization was performed by the R package of “ggplot2” and GraphPad Prism (Version 8).

## Figures and Tables

**Table 1:** The HADDOCK and HDOCK predicted docking scores for wild type-NTD-4A8 and Delta-NTD-4A8 complexes.

Docking parameter	Wild type-NTD-4A8	Delta-NTD-4A8
HADDOCK score	−84.4 ± 8.2	−65.0 ± 5.7
Cluster size	11	9
RMSD from the overall lowest-energy structure	0.5 ± 0.4	20.4 ± 2.4
Van der Waals energy	−60.3 ± 5.1	−47.1 ± 4.3
Electrostatic energy	−163.1 ± 9.1	−172.4 ± 24.3
Desolvation energy	−38.8 ± 1.1	−23.2 ± 3.5
Restraints violation energy	473.3 ± 42.2	397.4 ± 65.7
Buried surface area	1724.4 ± 44.2	1643.9 ± 47.6
*Z*-score	-2.6	-2.1
HDOCK docking score	-237.21	-237.07

Notes: additional parameters, including cluster size, RMSD from the overall lowest-energy structure, van der Waals energy, electrostatic energy, desolvation energy, restraints violation energy, buried surface area, and *Z*-score, are presented. The scores for other mutant complexes are shown in Table S2.

**Table 2:** The HADDOCK and HDOCK predicted docking scores for wild type-RBD-ACE2 and Delta-RBD-ACE2 complexes.

Docking parameter	Wild type-RBD-ACE2	Delta-RBD-ACE2
HADDOCK score	−116.1 ± 1.6	−102.7 ± 4.3
Cluster size	119	55
RMSD from the overall lowest-energy structure	6.5 ± 0.2	1.7 ± 1.4
Van der Waals energy	−56.4 ± 3.0	−35.6 ± 4.8
Electrostatic energy	−221.5 ± 11.0	−264.5 ± 23.9
Desolvation energy	−17.7 ± 2.5	−18.6 ± 5.0
Restraints violation energy	22.1 ± 16.6	44.0 ± 19.0
Buried surface area	1710.8 ± 41.9	1553.8 ± 106.5
*Z*-score	-1.3	-1.8
HDOCK docking score	-310.19	-240.73

Notes: additional parameters, including cluster size, RMSD from the overall lowest-energy structure, van der Waals energy, electrostatic energy, desolvation energy, restraints violation energy, buried surface area, and *Z*-score, are presented. The scores for other mutant complexes are shown in Table S3.

**Table 3:** The HADDOCK predicted docking scores for wild type-RBD-S2H14 and Delta-RBD-S2H14 complexes.

Docking parameter	Wild type-RBD-S2H14	Delta-RBD-S2H14
HADDOCK score	−79.4 ± 3.7	−72.8 ± 1.4
Cluster size	16	24
RMSD from the overall lowest-energy structure	8.8 ± 1.4	21.1 ± 0.4
Van der Waals energy	−40.7 ± 2.5	−44.9 ± 2.4
Electrostatic energy	−141.0 ± 22.6	−136.6 ± 13.0
Desolvation energy	−11.4 ± 7.3	−1.2 ± 2.6
Restraints violation energy	9.6 ± 15.6	7.0 ± 11.8
Buried surface area	1186.3 ± 105.6	1182.4 ± 42.8
*Z*-score	-1.8	-1.8

Notes: the scores for other mutant complexes are shown in Table S4. The protein-protein docking is based on the interactions between the heavy chain of the S2H14 antibody and S1 RBD.

**Table 4:** Virtual screening of potential drugs targeting Delta NTD, RBD, and RBM domains based on zinc database. Affinitive values of the top 10 best drugs are presented related to Table S5.

Targeted domains	ZINC ID	Drug name	Mwt	Values (kcal/Mol)
Delta NTD	ZINC30726863	Cepharanthine	606.719	-10.6
	ZINC100013130	Midostaurin	570.649	-10
	ZINC1539579	Targretin	348.486	-9.9
	ZINC14880001	ZINC14880001	528.537	-9.9
	ZINC14880002	Dihydroergotoxine	583.689	-9.8
	ZINC169289767	Trypan blue	872.894	-9.8
	ZINC3925861	Vorapaxar	492.591	-9.8
	ZINC52955754	Ergotamine	581.673	-9.7
	ZINC27990463	Lomitapide	693.732	-9.7
	ZINC3781738	Lestaurtinib	439.471	-9.6

Delta RBM	ZINC169289767	Trypan blue	872.894	-9.9
	ZINC4099104	Sn38 glucuronide	568.535	-9.6
	ZINC14880002	Dihydroergotoxine	583.689	-9.6
	ZINC1612996	Irinotecan	586.689	-9.4
	ZINC3932831	Avodart	528.537	-9.4
	ZINC14880001	ZINC14880001	528.537	-9.4
	ZINC3978005	Dihydroergotamine	583.689	-9.3
	ZINC95618827	ZINC95618827	721.646	-9.1
	ZINC13444037	Tasosartan	411.469	-9.1
	ZINC3978083	Tubocurarin	609.743	-9.1

Delta RBD	ZINC169289767	Trypan blue	872.894	-9.8
	ZINC4099104	Sn38 glucuronide	568.535	-9.5
	ZINC14880002	Dihydroergotoxine	583.689	-9.5
	ZINC14880001	ZINC14880001	528.537	-9.4
	ZINC1612996	Irinotecan	586.689	-9.3
	ZINC3932831	Avodart	528.537	-9.3
	ZINC3978083	Tubocurarin	609.743	-9.2
	ZINC3978005	Dihydroergotamine	583.689	-9.2
	ZINC13444037	Tasosartan	411.469	-9.1
	ZINC100378061	Naldemedine	570.646	-9

## Data Availability

All the data is available on RCSB and UniProt, and any simulation data will be provided on demand. The protein structural files predicted by AlphaFold were submitted as supplemental materials. The RStudio code used in this study to perform statistical analysis and visualize data is available upon request.

## Supplementary Materials

Supplementary Materials Figure S1: diversities of mutated nucleotides on SARS-CoV-2 genome. Figure S2: analysis of amino acid mutation on spike protein in different SARS-CoV-2 strains. (A) A structural illustration of the spike protein of SARS-CoV-2. The residue numbers of each region correspond to their positions in the spike protein of SARS-CoV-2. (B) The aligned spike protein's sequences of eleven SARS-CoV-2 strains. Figure S3: the comparisons of the full-length spike protein of the SARS-CoV-2 strains. Figure S4: the comparisons of S1 NTD of ten SARS-CoV-2 strains. Figure S5: the comparisons of S1 RBD of ten SARS-CoV-2 strains. Figure S6: percentage of the amino acid residues whose pLDDT is more than 70 in groups of S1 NTD, S1 RBD, and spike protein. Table S1: the cluster by spike protein structures. Table S2: docking analyses of antibody 4A8 with S1 NTD of different SARS-CoV-2 variants. Table S3: docking analyses of ACE2 with S1 RBD of different SARS-CoV-2 variants. Table S4: docking analyses of antibody S2H14 with S1 RBD of different SARS-CoV-2 variants. Table S5: virtual screening of potential drugs. Protein structural files of all SARS-CoV-2 variants were submitted as supplemental materials.
