# Supplementary material for: Structural Comparison and Drug Screening of Spike Proteins of Ten SARS-CoV-2 Variants
Source: Research (Wash D C). 2022 Feb 1;2022:9781758. doi: 10.34133/2022/9781758 (PMC8829538; doi:10.34133/2022/9781758)
Supplement: Supplementary Materials — Figure S1: diversities of mutated nucleotides on SARS-CoV-2 genome. Figure S2: analysis of amino acid mutation on spike protein in different SARS-CoV-2 strains. (A) A structural illustration of the spike protein of SARS-CoV-2. The residue numbers of each region correspond to their positions in the spike protein of SARS-CoV-2. (B) The aligned spike protein's sequences of eleven SARS-CoV-2 strains. Figure S3: the comparisons of the full-length spike protein of the SARS-CoV-2 strains. Figure S4: the comparisons of S1 NTD of ten SARS-CoV-2 strains. Figure S5: the comparisons of S1 RBD of ten SARS-CoV-2 strains. Figure S6: percentage of the amino acid residues whose pLDDT is more than 70 in groups of S1 NTD, S1 RBD, and spike protein. Table S1: the cluster by spike protein structures. Table S2: docking analyses of antibody 4A8 with S1 NTD of different SARS-CoV-2 variants. Table S3: docking analyses of ACE2 with S1 RBD of different SARS-CoV-2 variants. Table S4: docking analyses of antibody S2H14 with S1 RBD of different SARS-CoV-2 variants. Table S5: virtual screening of potential drugs. Protein structural files of all SARS-CoV-2 variants were submitted as supplemental materials. [file 9781758.f1.zip › supplemental figures-2021-12-22.docx]

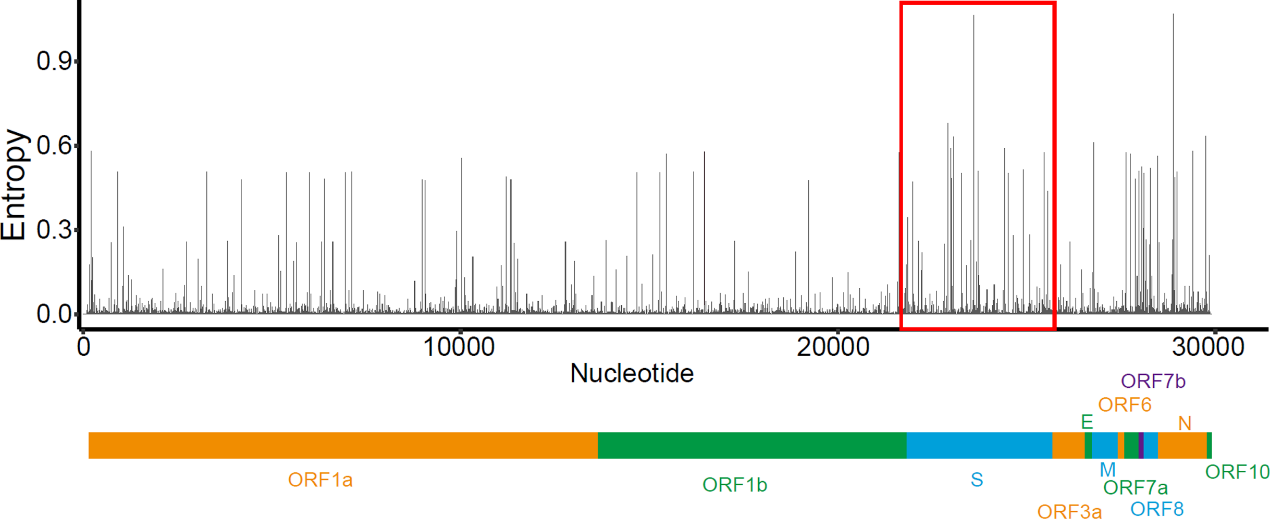


Figure S1. Diversities of mutated nucleotides on SARS-CoV-2 genome. The frequencies of genomic mutated nucleotides are shown in the up panel. The data of the diversity panel showing normalized Shannon entropy per codon are downloaded from GISAID (nextstrain.org/SARS-CoV-2). Genomic structure and gene location are shown in the down graphics. The red box indicates the high mutated frequency on spike protein.


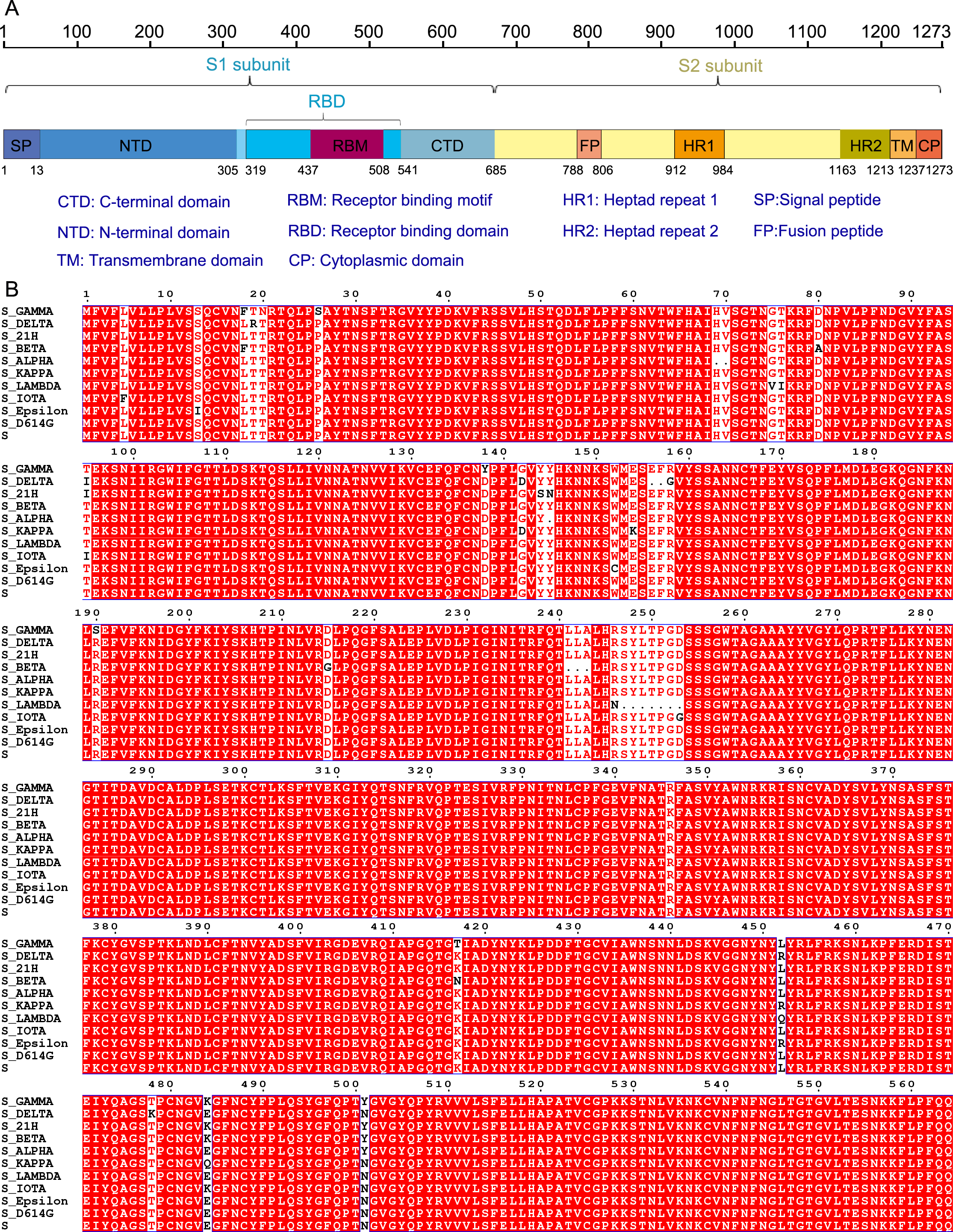

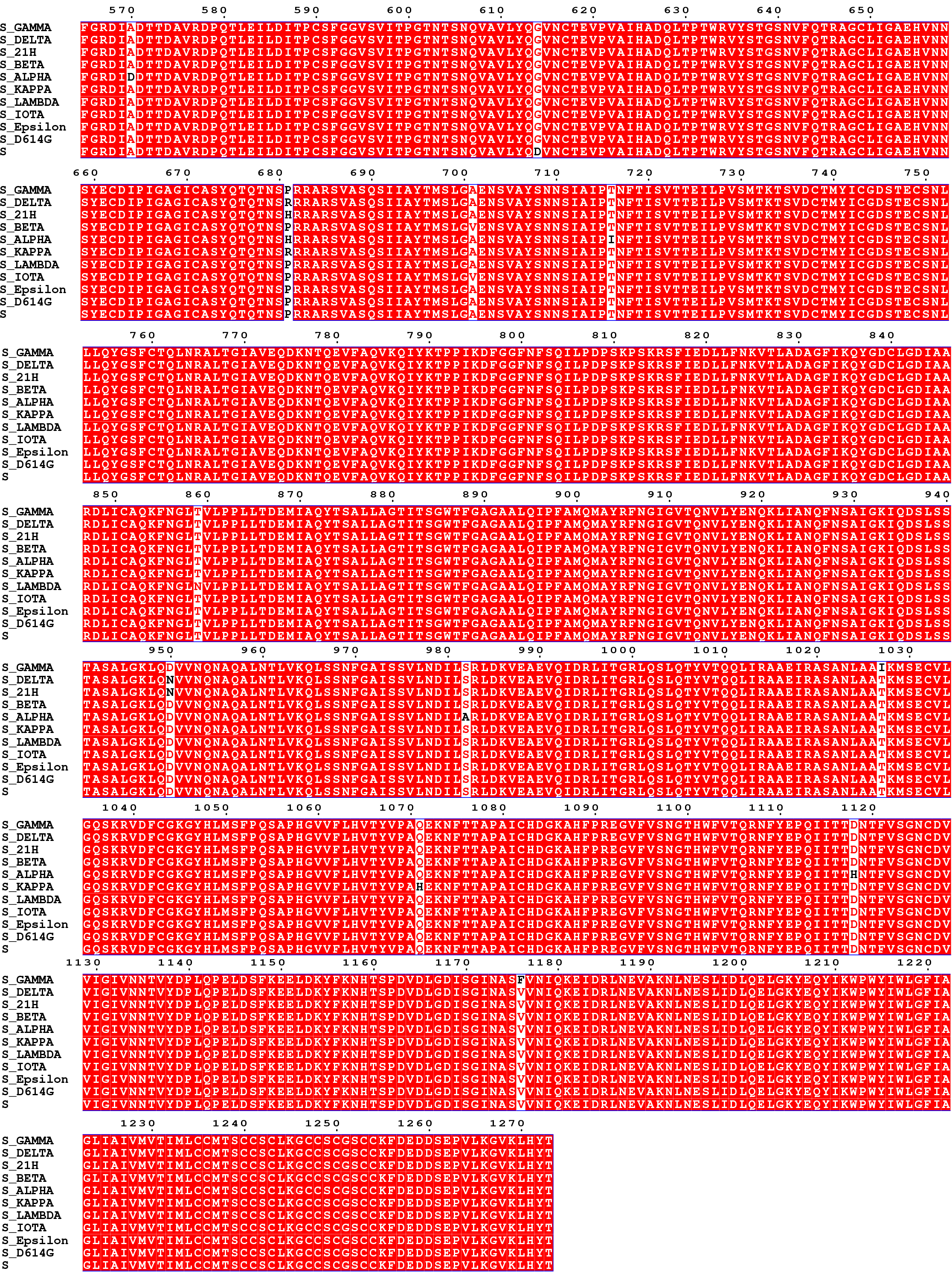


Figure S2. Analysis of amino acid mutation on spike protein in different SARS-CoV-2 strains. (A) A structural illustration of the spike protein of SARS-CoV-2. The residue numbers of each region correspond to their positions in the spike protein of SARS-CoV-2. (B) The aligned spike protein’s sequences of eleven SARS-CoV-2 strains.


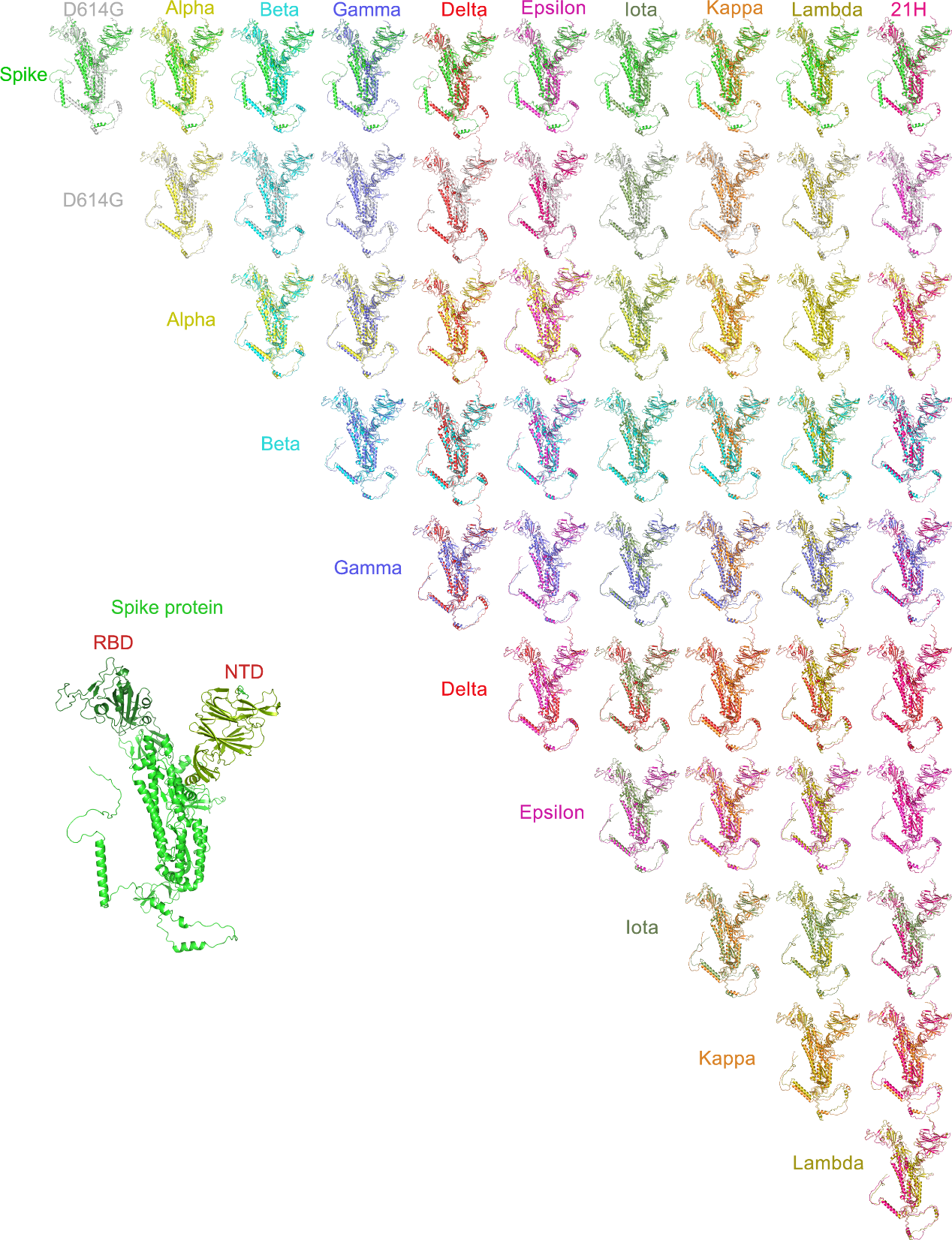


Figure S3. The comparisons of the full-length spike protein of the SARS-CoV-2 strains. Eleven strains are presented, including D614G mutant, Original, Alpha, Beta, Gamma, Delta, Epsilon, Iota, Kappa, Lambda, and 21H strains. The structures of different strains are displayed in different colors.


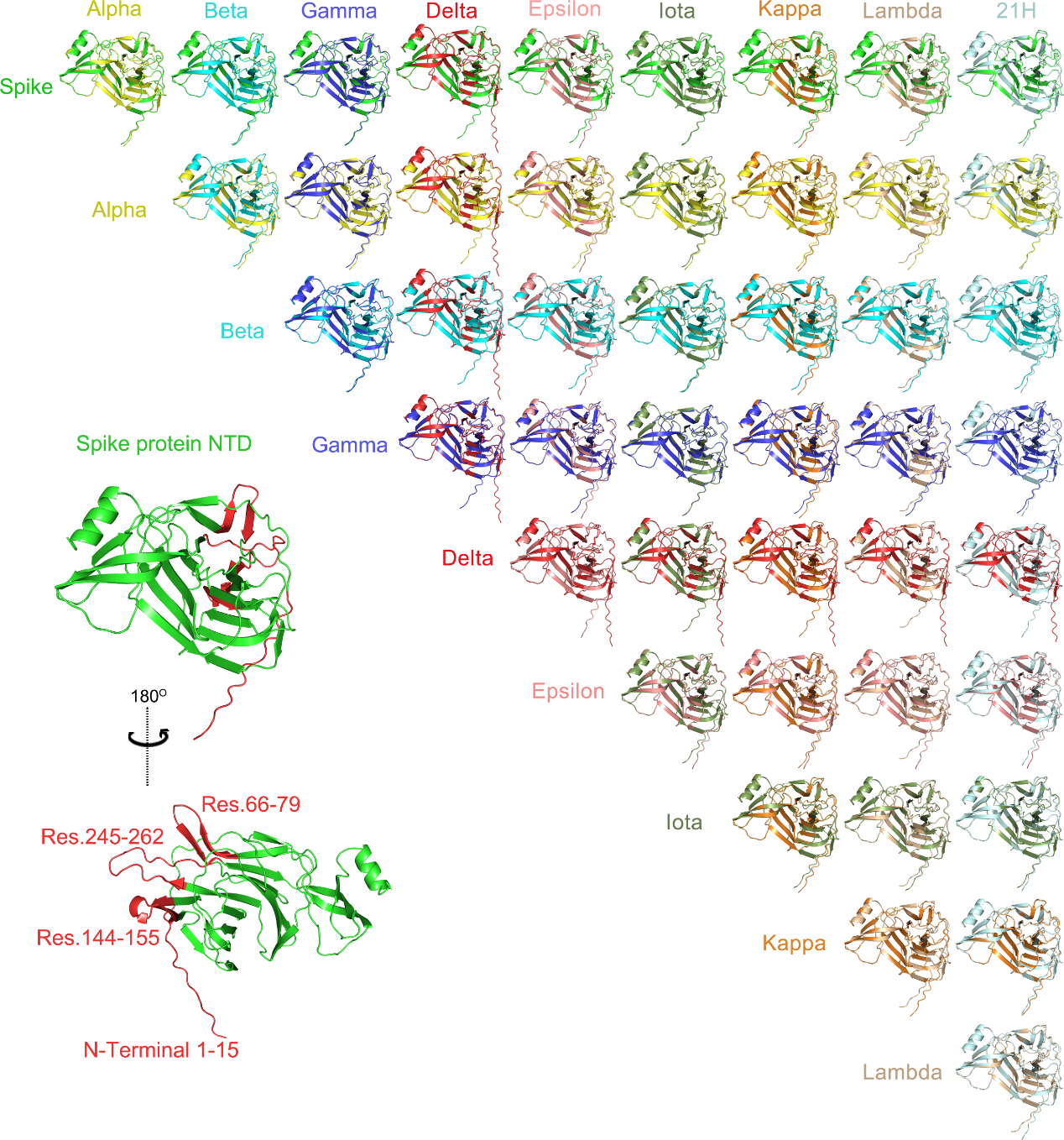


Figure S4. The comparisons of S1 NTD of ten SARS-CoV-2 strains. The structures of different strains are displayed in different colors.


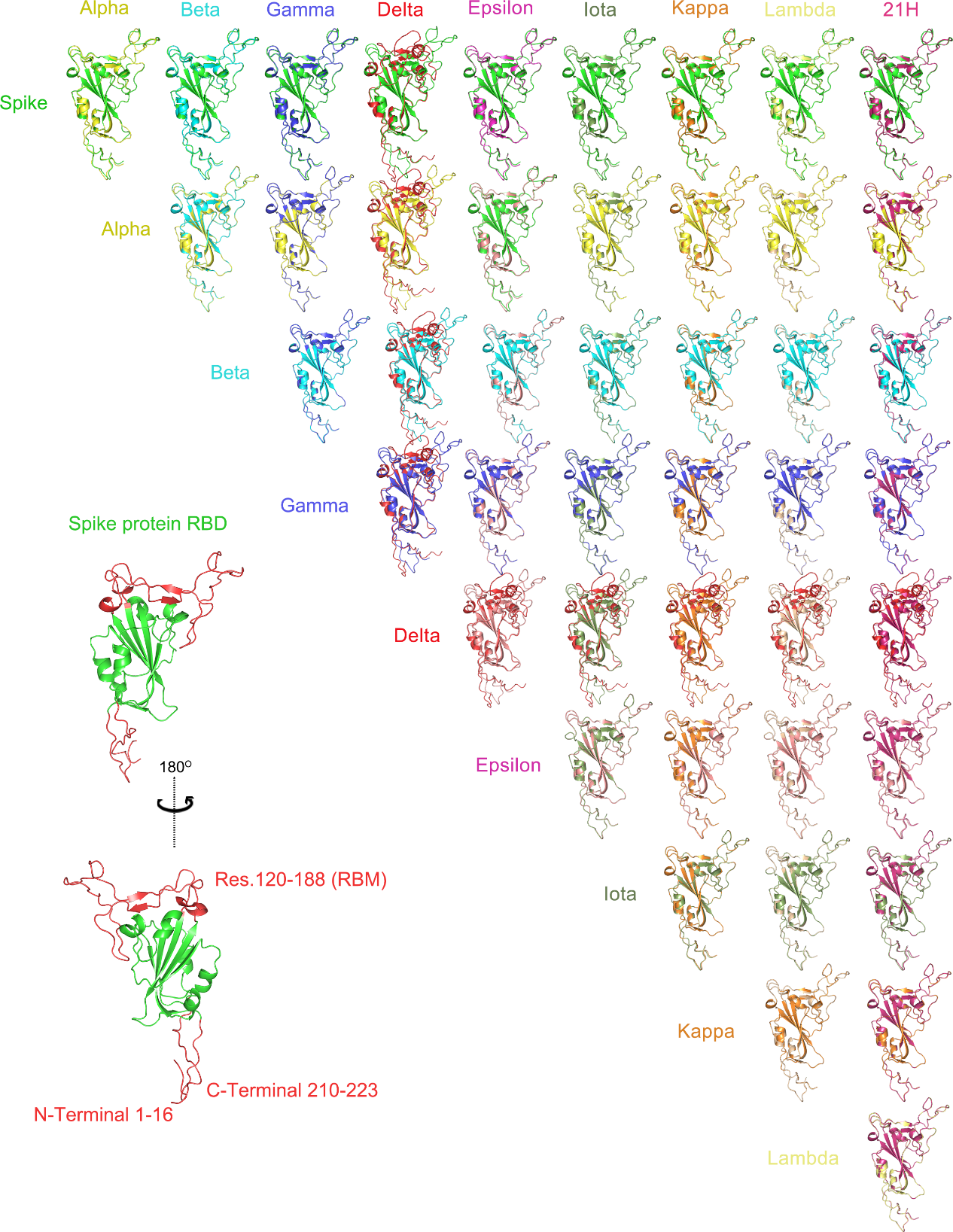


Figure S5. The comparisons of S1 RBD of ten SARS-CoV-2 strains. The structures of different strains are displayed in different colors.


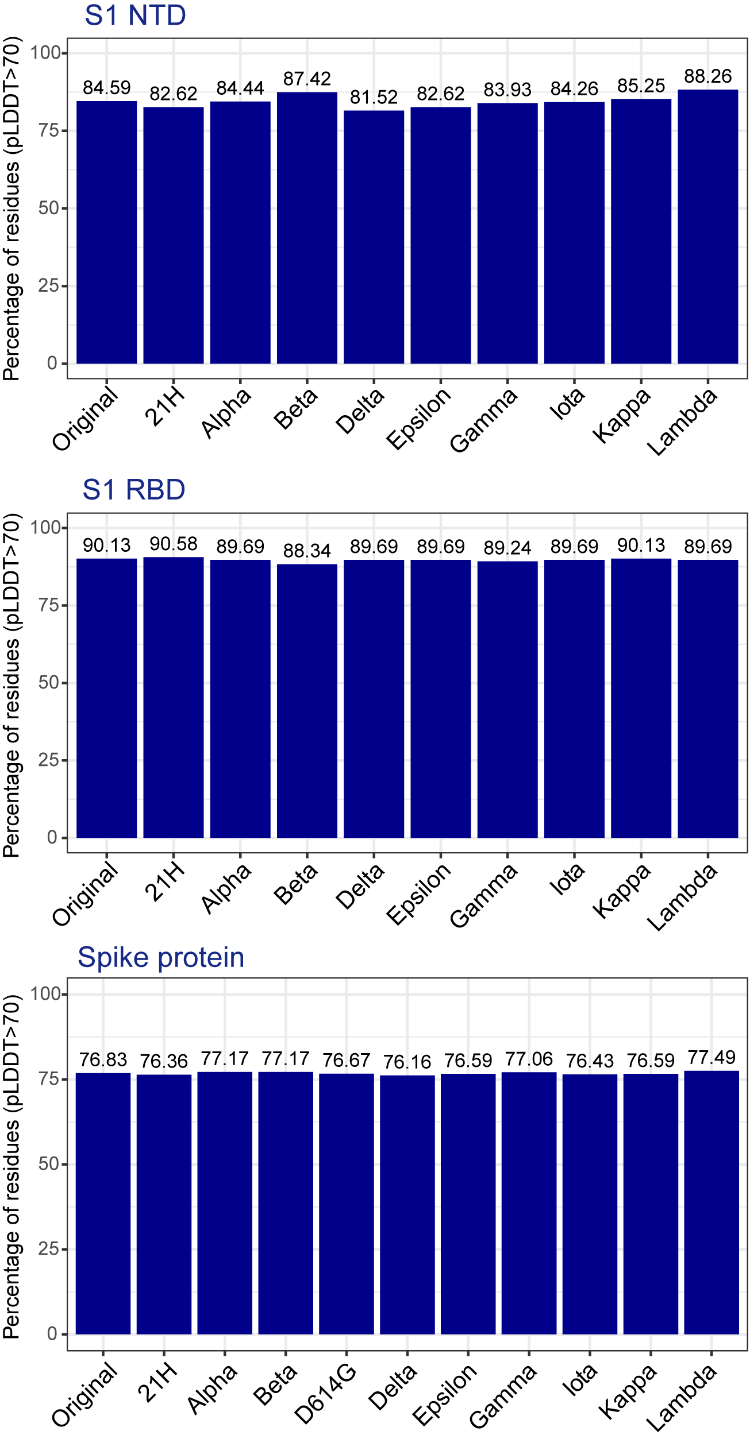


Figure S6. Percentage of the amino acid residues whose pLDDT is more than 70 in groups of S1 NTD, S1 RBD, and spike protein.
